# Elevated highly sensitive C-reactive protein in fibromyalgia associates with symptom severity

**DOI:** 10.1093/rap/rkac053

**Published:** 2022-06-25

**Authors:** Teemu Zetterman, Ritva Markkula, Eija Kalso

**Affiliations:** Pain Clinic, Department of Anaesthesiology, Intensive Care and Pain Medicine, Helsinki University and Helsinki University Hospital, Helsinki; City of Vantaa Health Centre, Vantaa; Department of General Practice and Primary Health Care; Pain Clinic, Department of Anaesthesiology, Intensive Care and Pain Medicine, Helsinki University and Helsinki University Hospital, Helsinki; Pain Clinic, Department of Anaesthesiology, Intensive Care and Pain Medicine, Helsinki University and Helsinki University Hospital, Helsinki; SLEEPWELL Research Programme, Faculty of Medicine, University of Helsinki, Helsinki, Finland

**Keywords:** FM, inflammation, CRP, hsCRP, widespread pain, chronic pain

## Abstract

**Objectives:**

Fibromyalgia (FM), a common pain syndrome, is thought to be a non-inflammatory, nociplastic condition, but evidence implicating neuroinflammation has been increasing. Systemic inflammation may be associated with more severe symptoms in some FM patients. We studied healthy controls and FM patients with and without systemic inflammation detectable using high-sensitivity CRP (hsCRP) measurement.

**Methods:**

We measured hsCRP levels and gathered clinical and questionnaire data [including the Fibromyalgia Impact Questionnaire (FIQ)] from 40 female FM patients and 30 age-matched healthy women. An hsCRP level >3 mg/l was considered elevated.

**Results:**

FM patients had significantly higher mean hsCRP levels than controls, explained by overweight and lower leisure-time physical activity. Eight FM patients had elevated hsCRP levels and 29 had normal hsCRP levels. Levels of hsCRP were significantly correlated with FIQ scores. Patients with elevated hsCRP had higher FIQ scores, with worse physical functioning and greater pain and were less likely to be employed than patients with normal hsCRP. These patient groups did not differ by blood count, liver function or lipid profiles, nor by education, psychological measures, sleep disturbance, smoking or comorbidities.

**Conclusion:**

Some FM patients have elevated hsCRP, mostly due to overweight and physical inactivity. They have worse symptoms and their ability to work is impaired. Measurement of hsCRP may help to identify FM patients in greatest need of interventions supporting working ability.

**Trial registration:**

ClinicalTrials.gov (https://clinicaltrials.gov), NCT03300635

Key messages

- Elevated highly sensitive C-reactive protein (hsCRP) is associated with worse fibromyalgia symptoms.
- Elevated hsCRP in fibromyalgia is mostly explained by overweight and physical inactivity.
- Assessment of inflammation may help to identify fibromyalgia patients who would benefit from more intensive treatment interventions.

## Introduction

Fibromyalgia (FM) was historically considered to be a rheumatic condition initiated by inflammation [1]. As no inflammation has been identified, FM is now classified as a nociplastic condition where pain symptoms are caused by functional changes in the CNS [2–4].

Over time, however, evidence has accumulated to suggest some immunological component in FM. FM patients have been reported to have higher cerebrospinal fluid (CSF) and serum levels of pro-inflammatory cytokines IL-6, IL-8 and TNF-α and lower levels of anti-inflammatory IL-10 than controls [5–8]. Systemic inflammation may be upregulated by the CNS. Elevated levels of substance P and neurotrophic factors have been reported in the CSF of FM patients [9, 10]. Substance P and neurotrophic factors activate microglia, which can lead to neuroinflammation and heightened nociception and possibly mediate between psychosocial stress and somatic symptoms [8].

CRP is an acute phase protein produced mainly by liver hepatocytes in response to IL-6 and is a non-specific marker of inflammation that is widely used in, for example, evaluating the severity of inflammation and clinically differentiating between bacterial and viral infections [11]. CRP is also slightly elevated in many chronic conditions, including pulmonary or heart disease, diabetes and osteoporosis [12, 13]. High BMI raises CRP levels through low-grade inflammation in adipose tissue [13, 14]. Chronically elevated CRP is also a risk factor for atherosclerosis and cardiovascular events [15]. As CRP is usually markedly elevated only in acute inflammation or infection, high-sensitivity CRP (hsCRP) measurement is useful in low-grade and chronic conditions, as it allows accurate measurement at low levels [16].

In some studies, elevated hsCRP has been associated with depression [17, 18], anxiety [18] and perceived stress [17], but not in others [17, 19]. Previous studies have also found hsCRP to be elevated in some FM patients [12, 20–22]. Pérez-Aranda *et al.* [23] identified four distinct clusters of FM patients, with one cluster (33% of their sample) characterized by elevated hsCRP and the most severe symptoms.

Here we seek to confirm whether FM patients have higher hsCRP than controls and whether FM symptom severity and clinical characteristics differ with hsCRP levels. We hypothesized that FM patients would have higher mean hsCRP levels and levels of hsCRP would correlate with the severity of FM symptoms.

## Methods

### Patients

This work is a part of our larger study of metabolism and muscle function in FM. For that we recruited during our funding period the most patients and age- and gender-matched volunteers available from Helsinki University Hospital outpatient clinics and primary health care. These were 51 female FM patients ages 18–65 years and 31 age-matched healthy women.

The subjects had participated in a cognitive stress test with surface electromyography and electrocardiography between November 2015 and June 2018 [24]. During these sessions we collected medical histories and background, lifestyle and questionnaire data. Subjects were invited to participate in an oral glucose tolerance test, with 41 patients and 30 controls attending (reported previously) [25]. The diagnosis of FM was based on American College of Rheumatology 1990 criteria [3].

The study was approved by the Ethics Committee of the Helsinki and Uusimaa Hospital District and retrospectively registered in ClinicalTrials.gov (NCT03300635) on 3 October 2017. All study subjects gave written informed consent.

### Background and lifestyle data and medical histories

We collected data on the subjects’ backgrounds and medical histories using questionnaires and clinical interviews, recording weight, height, BMI, education, working status and smoking status. Leisure-time physical activity (LTPA) was rated for frequency (1, not at all; 2, 1–2 times per month; 3, 1–2 times per week; 4, several times per week) and intensity (1, walking; 2, intermittent walking/jogging; 3, slow running/jogging; 4, running). Study subjects subjectively rated their physical fitness in comparison with healthy peers as worse, average or better on a scale of 1–3, respectively. We summed these for an overall score (3–11), dichotomizing subjects to two LPTA categories: active (≥8) and inactive (<8). Sleep disturbance (yes/no) and waking during sleep (none, 1–2 times, 3–4 times or ≥5 times per night) were recorded, as were other chronic diagnoses.

### Questionnaires

The 10-item Fibromyalgia Impact Questionnaire (FIQ) measures the severity of FM symptoms and their impact on daily functions during the previous 7 days [26], with the score ranging from 0 (no symptoms/impact) to 100 (greatest symptoms/impact). The items, each standardized to 0–10, are 1) ability to do daily activities, with 10 subitems (e.g. cooking, cleaning, visiting friends/family), rated on a Likert scale of 0 (always)–3 (never); 2) number of days feeling good; 3) number of days of missed work; 4) interference with ability to work; 5) pain severity; 6) tiredness; 7) unrefreshing sleep; 8) stiffness; 9) nervousness/anxiety; 10) depression. Items 4–10 are rated on a 10 cm visual analogue scale (VAS). We used the validated Finnish translation (Finn-FIQ) of the 1991 version [27].

The FM patients were assessed against ACR1990 criteria, as we believed they would result in the most uniform patient group. We included the revised ACR 2016 criteria to increase comparability between our work and other studies [4]. These comprise the Widespread Pain Index (WPI), rated from 0 to 19 (1 point for each of 19 possible anatomical pain sites) and the Symptom Severity Scale (SSS). This rates the severity of fatigue, unrefreshing sleep and cognitive symptoms on a Likert scale of 0 (none)–3 (severe and constant) and the presence of headaches, depression and lower abdominal pain (0, no; 1, yes). SSS scores range from 0 to 12. The ACR 2016 criteria for FM are met when WPI is ≥7 and SSS is ≥5 or WPI is ≥3 and SSS is ≥9, with the requirement that symptoms persist for ≥3 months.

The Pain Catastrophizing Scale (PCS) rates 13 items, e.g. ‘I worry whether my pain will ever cease’ on a Likert scale of 0 (never)–4 (constantly) for a total of 0–52 (maximum catastrophizing) [28].

The State-Trait Anxiety Inventory B (STAI-B) was used to measure the more stable trait of anxiety, i.e. the tendency for anxiety. STAI-B contains 20 items describing how the subject usually feels (e.g. ‘I feel calm and composed’ or ‘I worry too much over unimportant things’), each rated on a Likert scale of 1 (not at all)–4 (very much) for a total of 20–80, with a greater score indicating more trait anxiety. We used the STAI-X version [29].

### Blood samples

Blood samples were collected between December 2015 and February 2019 at the Helsinki University Hospital laboratories (HUSLAB) by HUSLAB staff. Subjects were to discontinue non-essential medication, avoid strenuous physical activity the previous day and fast 10–12 h before collection. Samples were taken (0700–0900) from the cubital vein and analysed within 3 h immunoturbimetrically [Roche Modular with reagent Tina-quant Cardiac CRP (Latex) High Sensitive assay, catalogue #11972855, Roche Diagnostics (Basel, Switzerland) between December 2015 and May 2016, and Architect c8000 with reagent Multigent CRP Vario, catalogue #6K26-30, Abbott Laboratories (Abbott Park, IL, USA) between March 2016 and February 2019].

The cut-off values for elevated hsCRP vary in the FM literature [12, 20–22]. The 90th centile limit for CRP in healthy young adults is 3 mg/l [30]. Thus we considered >3 mg/l hsCRP to be elevated.

The following were also determined from the fasting venous blood sample: blood haemoglobin (Hb, g/l), blood haematocrit (%), blood erythrocyte count (× 10^12^/l), mean cellular volume of erythrocytes (fl), erythrocyte distribution width (%), mean corpuscular haemoglobin (MCH, pg/cell), mean corpuscular haemoglobin concentration (MCHC, g/l), blood leucocyte count (× 10^9^/l), blood thrombocyte count (× 10^9^/l), plasma activated partial thromboplastin time (sec), international normalized ratio, plasma creatine kinase (U/l), plasma total cholesterol (mmol/l), plasma high-density lipoprotein (HDL, mmol/l), plasma triglycerides (TGs, mmol/l), plasma low-density lipoprotein (LDL, mmol/l), fasting glucose (mmol/l), fasting plasma lactate (mmol/l) and fasting blood pyruvate (µmol/l). From the ninth FM patient and the eighth control, the following were also analysed: serum pH, actual serum ionized calcium (mmol/l), serum ionized calcium normalized to pH 7.4, plasma alkaline phosphatase (U/l), plasma alanine transaminase (U/l), plasma aspartate transaminase (U/l), serum ANA (titre), serum extractable nuclear antigens (yes/no) and serum glutamate decarboxylase antibodies (IU/ml).

### Statistics

We identified extreme outliers using the boxplot method. We tested normality of hsCRP data with the Shapiro–Wilk test (*P* > 0.05), logarithmically transformed if non-normal. We used Levene’s test to determine the homogeneity of variance.

We used a multivariate linear model to adjust for the effect of lifestyle factors (e.g. BMI, LTPA, smoking, sleep disturbance) on hsCRP differences between FM patients and controls. We tested univariate models for lifestyle factors (e.g. hsCRP, BMI). Variables with possible associations at *P* < 0.1 were added with the group variable into the final model. To detect multicollinearity, we calculated variance inflation factors for the included explanatory factors.

We expected hsCRP to correlate positively with FIQ, STAI-B and PCS scores and with glucose, the area under the curve (AUC) for glucose, LDL and TGs, but negatively with HDL. We calculated Pearson correlation coefficients between these measures or Spearman rank correlation coefficients when normality was not satisfied.

For comparisons between FM patients and controls and between FM patients with elevated or normal hsCRP, we used Student’s *t*-test for continuous variables and Fisher’s exact test for categorical variables. We considered two-tailed *P*-values <0.05 to be significant.

## Results

Two FM patients were excluded from the analyses, one due to her being diagnosed with diabetes in the oral glucose tolerance test and one not having hsCRP data. We identified three extreme hsCRP outliers: two FM patients (hsCRP 15.01 and 22.71 mg/l) and one control (hsCRP 10.50 mg/l). While the other FM patients had a history of gout, the two other outliers had no chronic inflammatory condition. Although subjects had been instructed to postpone participation in the study in cases of acute illness, we decided that the three extreme outliers likely fell into this category and they were excluded from further analyses. Thus the final dataset comprised 37 FM patients and 29 controls.

FM patients and controls did not differ by age or smoking status, but the former had higher BMIs, were less physically active, were more likely to have other diagnoses, had lower education and were less likely to be working than controls (Table 1). Other diagnoses are presented in Supplementary Table S1, available at *Rheumatology Advances in Practice* online. FM patients had higher haemoglobin, leucocyte count (LC), glucose AUC, LDL and TGs than controls (Table 2).

**Table 1:** Demographic and questionnaire data for healthy controls, all FM patients, FM patients with normal hsCRP and FM patients with elevated hsCRP

Characteristics	Control (*n* = 29)	FM (*n* = 37)	FM (normal hsCRP) (*n* = 29)	FM (elevated hsCRP) (*n* = 8)
Age (years)				
Mean (s.d.)	45.2 (11.6)	46.2 (11.3)	45.7 (11.2)	48.0 (12.3)
Median (minimum–maximum)	47.0 (22.0–61.0)]	47.0 (23.0–65.0)	47.0 (23.0–65.0)	52.5 (25.0–62.0)
*P*-value	0.739		0.636	
BMI (kg/m^2^)				
Mean (s.d.)	24.6 (3.29)	27.6 (5.86)	27.0 (5.07)	29.8 (8.09)
Median (minimum–maximum)	24.4 (19.1–32.2)	26.3 (19.3–45.4)	25.7 (20.2–37.2)	29.1 (19.3–45.4)
Missing, *n* (%)	1 (3.4)	1 (2.7)	1 (3.4)	0 (0)
*P*-value	**0.012**		0.374	
BMI category, *n* (%)				
Normal weight (18.5–24.9 kg/m^2^)	16 (55.2)	13 (35.1)	11 (37.9)	2 (25.0)
Overweight (25–30 kg/m^2^)	10 (34.5)	10 (27.0)	7 (24.1)	3 (37.5)
Obese (>30 kg/m^2^)	2 (6.9)	13 (35.1)	10 (34.5)	3 (37.5)
Missing	1 (3.4)	1 (2.7)	1 (3.4)	0 (0)
*P*-value	**0.022**		0.878	
Education (years beyond primary school)				
Mean (s.d.)	8.40 (2.04)	4.56 (3.10)	4.75 (3.26)	3.88 (2.53)
Median (minimum–maximum)	9.00 (3.00–13.0)	4.25 (0–13.0)	4.75 (0–13.0)	3.00 (1.00–8.00)
Missing, *n* (%)	0 (0)	1 (2.7)	1 (3.4)	0 (0)
*P*-value	**<0.001**		0.434	
Working, *n* (%)				
No	0 (0)	15 (40.5)	7 (24.1)	8 (100)
Yes	29 (100)	20 (54.1)	20 (69.0)	0 (0)
Missing	0 (0)	2 (5.4)	2 (6.9)	0 (0)
*P*-value	**<0.001**		**<0.001**	
Smoking, *n* (%)				
Non-smoker	27 (93.1	29 (78.4)	23 (79.3)	6 (75.0)
Smoker	2 (6.9)	7 (18.9)	5 (17.2)	2 (25.0)
Missing	0 (0)	1 (2.7)	1 (3.4)	0 (0)
*P*-value	0.172		0.639	
LTPA score (3–11)				
Mean (s.d.)	8.31 (1.81)	6.86 (1.75)	6.89 (1.83)	6.71 (1.50)
Median (minimum–maximum)	9.00 (4.00–11.0)	7.00 (4.00–11.0)	7.00 (4.00–11.0)	7.00 (5.00–9.00)
Missing, *n* (%)	0 (0)	2 (5.4)	1 (3.4)	1 (12.5)
*P*-value	**0.002**		0.793	
LTPA category, *n* (%)				
Inactive (LTPA score <8)	9 (31.0)	23 (62.2)	17 (58.6)	6 (75.0)
Active (LTPA score ≥8)	20 (69.0)	14 (37.8)	12 (41.4)	2 (25.0)
*P*-value	**0.015**		0.683	
Sleep problems, *n* (%)				
No	26 (89.7)	6 (16.2)	6 (20.7)	0 (0)
Yes	3 (10.3)	30 (81.1)	22 (75.9)	8 (100)
Missing	0 (0)	1 (2.7)	1 (3.4)	0 (0)
*P*-value	**<0.001**		0.302	
Number of comorbidities				
Mean (s.d.)	0.50 (0.66)	2.08 (1.81)	1.82 (1.68)	3.00 (2.07)
Median (minimum–maximum)	0 (0–2.00)	2.00 (0–7.00)	2.00 (0–5.00)	2.50 (1.00–7.00)
Missing, *n* (%)	5 (17.2)	1 (2.7)	1 (3.4)	0 (0)
*P*-value	**<0.001**		0.171	
Trait Anxiety Inventory (STAI-B)				
Mean (s.d.)	28.3 (5.43)	45.4 (9.51)	44.3 (9.65)	49.7 (8.14)
Median (minimum–maximum)	27.0 (21.0–44.0)	46.0 (29.0–64.0)	42.0 (29.0–64.0)	46.0 (41.0–64.0)
Missing, *n* (%)	1 (3.4)	3 (8.1)	2 (6.9)	1 (12.5)
*P*-value	**<0.001**		0.158	
PCS				
Mean (s.d.)		17.1 (10.1)	17.0 (9.14)	19.5 (13.5)
Median (minimum–maximum)		16.0 (0.0–48.0)	16.5 (0–40.0)	16.0 (7.00–48.0)
Missing		1 (2.7)	1 (3.4)	0 (0)
*P*-value			0.638	

Statistical testing between healthy controls and all FM patients and between FM patient subgroups was done with a *t*-test for continuous variables and Fisher’s test for categorical variables. *P*-values <0.05 in bold.

**Table 2:** Blood sample data for healthy controls, all FM patients, FM patients with normal hsCRP and FM patients with elevated hsCRP

	Control (*n* = 29)	FM (*n* = 37)	FM (normal hsCRP) (*n* = 29)	FM (elevated hsCRP) (*n* = 8)
Highly sensitive CRP (mg/l)				
Mean (s.d.)	1.17 (1.11)	2.33 (2.43)	1.19 (0.675)	6.46 (1.89)
Median (minimum–maximum)	0.680 (0.13–3.78)	1.51 (0.19–9.22)	1.12 (0.190–2.66)	6.46 (3.30–9.22)
*P*-value	**0.013**		**<0.001**	
Elevated hsCRP (>3 mg/l), *n* (%)				
No	26 (89.7)	29 (78.4)	29 (100)	0 (0)
Yes	3 (10.3)	8 (21.6)	0 (0)	8 (100)
*P*-value	0.323		**< 0.001**	
Haemoglobin (g/l)				
Mean (s.d.)	128 (7.59)	133 (7.77)	134 (7.09)	128 (8.74)
Median (minimum–maximum)	128 (105–145)	132 (115–153)	133 (121–153)	130 (115–140)
Missing	0 (0)	1 (2.7)	1 (3.4)	0 (0)
*P*-value	**0.021**		0.107	
Leucocyte count (× 10^9^/l)				
Mean (s.d.)	4.99 (1.10)	6.06 (1.77)	5.97 (1.64)	6.35 (2.27)
Median (minimum–maximum)	5.00 (3.00–8.80)	6.10 (3.00–10.8)	5.90 (3.20–10.0)	6.45 (3.00–10.8)
Missing, *n* (%)	0 (0)	1 (2.7)	1 (3.4)	0 (0)
*P*-value	**0.004**		0.67	
Thrombocyte count (× 10^9^/l)				
Mean (s.d.)	261 (41.8)	276 (65.5)	275 (62.9)	279 (78.7)
Median (minimum–maximum)	259 (196–342)	271 (170–469)	268 (176–469)	286 (170–392)
Missing, *n* (%)	0 (0)	1 (2.7)	1 (3.4)	0 (0)
*P*-value	0.264		0.907	
Glucose at 0 h (mmol/l)				
Mean (s.d.)	5.31 (0.501)	5.45 (0.535)	5.42 (0.538)	5.56 (0.542)
Median (minimum–maximum)	5.30 (4.30–6.40)	5.40 (4.50–6.80)	5.30 (4.50–6.80)	5.55 (4.70–6.30)
*P*-value	0.263		0.525	
Glucose AUC (mmol*h/l)				
Mean (s.d.)	11.4 (2.16)	13.7 (2.95)	13.8 (3.21)	13.1 (1.81)
Median (minimum–maximum)	11.2 (7.55–15.2)	13.1 (8.70–20.6)	13.7 (8.70–20.6)	12.6 (11.2–16.7)
Missing, *n* (%)	3 (10.3)	2 (5.4)	2 (6.9)	0 (0)
*P*-value	**0.001**		0.39	
Impaired glucose regulation, *n* (%)				
Yes	5 (17.2)	14 (37.8)	11 (37.9)	3 (37.5)
No	24 (82.8)	23 (62.2)	18 (62.1)	5 (62.5%)
*P*-value	0.1		1	
HDL (mmol/l)				
Mean (s.d.)	1.72 (0.370)	1.59 (0.376)	1.59 (0.343)	1.59 (0.509)
Median (minimum–maximum)	1.72 (1.05–2.65)	1.61 (0.900–2.47)	1.61 (0.900–2.29)	1.62 (0.920–2.47)
*P*-value	0.186		0.98	
LDL (mmol/l)				
Mean (s.d.)	2.74 (0.571)	3.42 (0.869)	3.38 (0.861)	3.59 (0.937)
Median (minimum–maximum)	2.70 (1.50–3.80)	3.40 (1.80–5.10)	3.30 (2.10–5.10)	3.65 (1.80–4.90)
*P*-value	**<0.001**		0.583	
TGs (mmol/l)				
Mean (s.d.)	0.974 (0.661)	1.28 (0.665)	1.27 (0.732)	1.34 (0.355)
Median (minimum–maximum)	0.770 (0.4403.73)	1.08 (0.530–3.64)	1.03 (0.530–3.64)	1.38 (0.820–1.76)
*P*-value	0.064		0.687	

Statistical testing between healthy controls and all FM patients and between FM patient subgroups was done with a *t*-test for continuous variables and Fisher’s test for categorical variables. *P-*values <0.05 in bold.

FM patients had higher hsCRP [mean 2.33 mg/l (s.d. 2.43) *vs* 1.17 (s.d. 1.11), *P* = 0.013) but no significant difference in the rate of elevated hsCRP (21.6% *vs* 10.3%, *P* = 0.323) compared with controls; hsCRP had homogeneous variance (Levene’s test, *P* = 0.063) but was non-normally distributed (Shapiro–Wilk test, *P* < 0.001). The natural logarithm of hsCRP (hsCRP_ln_) was normally distributed (Shapiro–Wilk test, *P* = 0.665) and had homogeneous variance (Levene’s test, *P* = 0.962), so we used hsCRP_ln_ for the linear model and correlation analyses.

In univariate models, group membership predicted hsCRP_ln_ (F[1, 64] = 6.818; *P* = 0.011; *R*^2^ = 0.0963). Of the adjusting factors, BMI predicted hsCRP_ln_ (F[1, 62] = 21.78, *P* < 0.001, *R*^2^ = 0.26), as did LTPA (F[1, 64] = 13.76, *P* < 0.001, *R*^2^ = 0.177) and sleep disturbance (F[1, 63] = 7.457, *P* = 0.008, *R*^2^ = 0.1058), and these were entered into the model. Smoking did not predict hsCRP_ln_ (F[1, 63] = 0.319, *P* = 0.574, *R*^2^ = 0.005308). There were no significant two-way interactions between groups, BMI, LTPA or sleep disturbance. In the model hsCRP_ln_ ∼ group + BMI + LTPA + sleep disturbance, sleep was no longer significant and removing it did not alter the fit of the model (Akaike information criterion 171.39 *vs* 171.18). Variance inflation factors were low for group membership (1.15), BMI (1.23) and LTPA (1.25), indicating no significant multicollinearity.

**Fig. 1:**
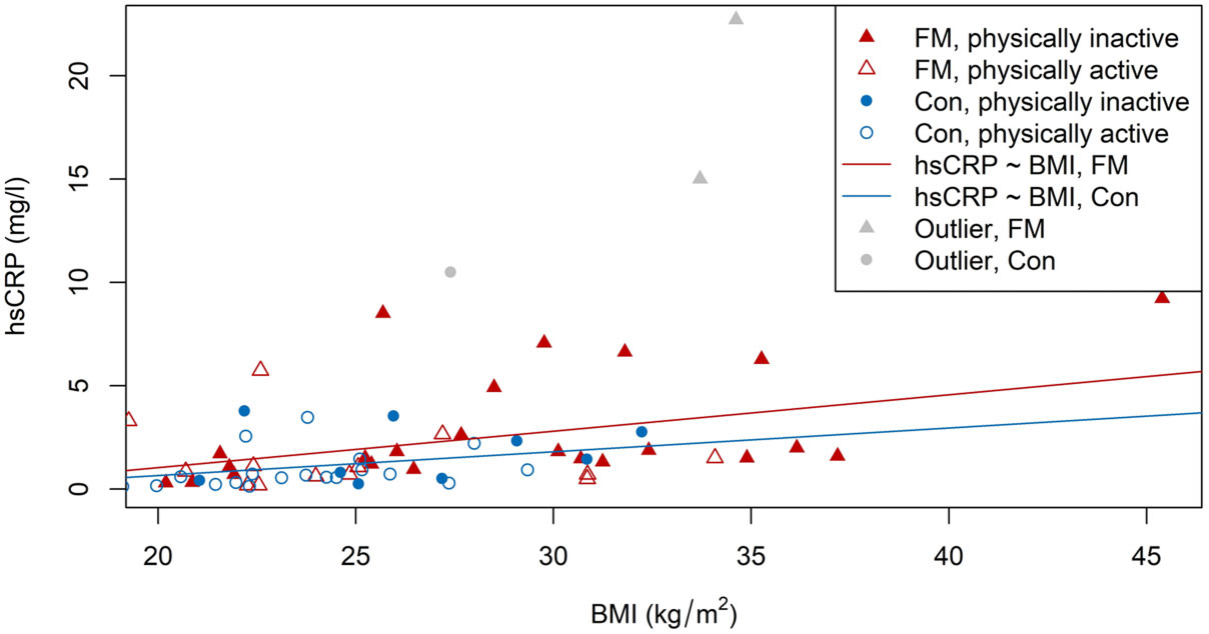
Relationship between BMI and hsCRP Regression lines are for linear regression between hsCRP and BMI for both FM patients and controls. Outliers not included in the regression are shown for completeness. con: control.

The final multivariate model was hsCRP_ln_ ∼ group + BMI + LTPA, which significantly predicted hsCRP_ln_ (F[3, 60] = 9.77, *P* < 0.001, *R*^2^ = 0.3282). Predicted hsCRP_ln_ was equal to −1.916 + 0.296 (group) + 0.0791 (BMI) − 0.442 (LTPA), where the group was coded as 0 = control or 1 = FM and LTPA was coded as 0 = inactive and 1 = active. After adjusting for BMI and LTPA, BMI was a significant predictor of hsCRP_ln_ (*P* = 0.002). LTPA was close to significance (*P* = 0.077), while group was no longer significant (*P* = 0.220). The relationship of BMI to hsCRP is visualized in Fig. 1.

**Fig. 2:**
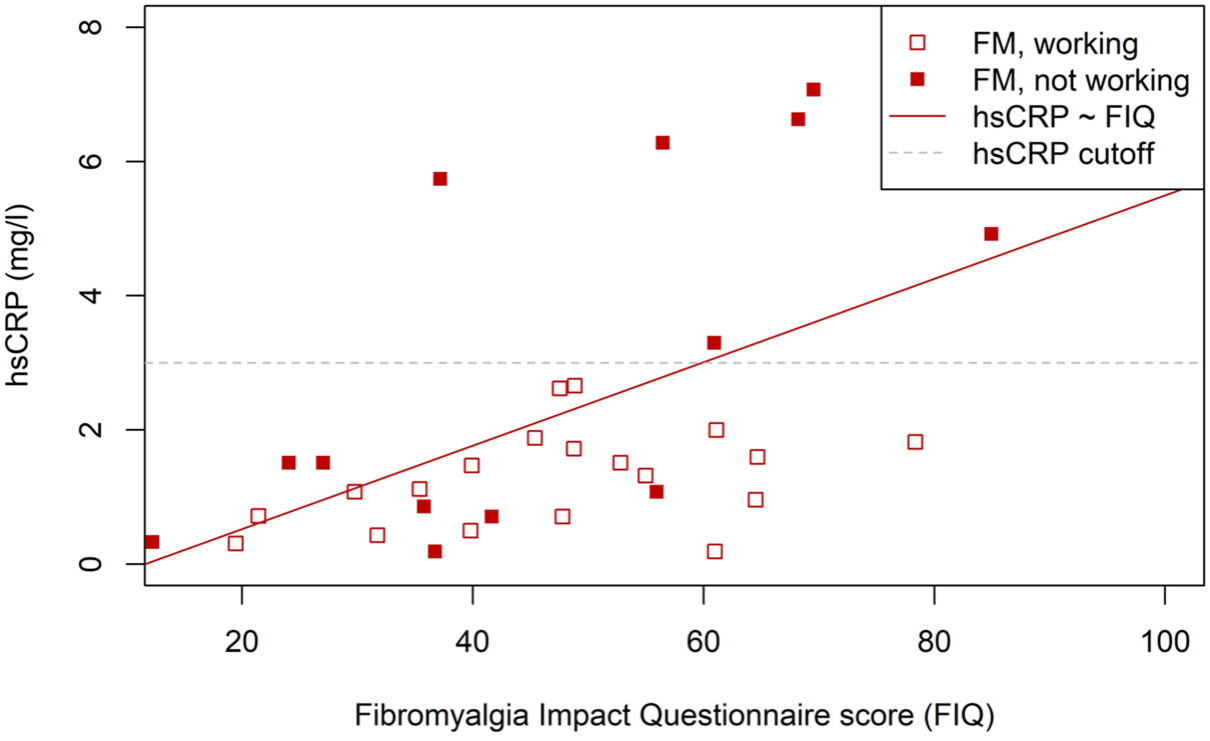
Relationship between hsCRP and symptom severity as measured by the FIQ score Regression line is for linear regression between hsCRP and FIQ. Outliers not included in the regression are shown for completeness.

We tested for correlations between hsCRP_ln_ and FIQ, STAI-B and PCS scores, and glucose, glucose AUC, HDL, LDL and TGs. None of these showed extreme outliers. All were normally distributed, apart from TGs (Shapiro–Wilk normality test, *P* < 0.001). We calculated Pearson correlation coefficients (or Spearman rank correlation coefficients for TGs). FIQ positively correlated with hsCRP_ln_ (r[34] = 0.532, *P* < 0.001) (Fig. 2). TGs also correlated with hsCRP_ln_ (ρ[36] = 0.370, *P* = 0.024). No other measures correlated significantly with hsCRP_ln_ (STAI-B ρ[32] = 0.162, *P* = 0.359; PCS ρ[34] = 0.194, *P* = 0.256; glucose ρ[35] = 0.167, *P* = 0.322; glucose AUC ρ[33] = 0.107, *P* = 0.542; HDL ρ[35] = −0.0047, *P* = 0.978; LDL ρ[35] = 0.201, *P* = 0.233).

Comparing the eight included FM patients with elevated hsCRP (21.6%) with the 29 FM patients with normal hsCRP (78.4%), the groups differed by likelihood of working (0% *vs* 69%, *P* < 0.001) and the elevated hsCRP group had higher FIQ scores [64.8 (s.d. 17.2) *vs* 45.5 (s.d. 17.4), *P* = 0.017] and, of individual FIQ items, reported worse physical functioning [4.33 (s.d. 2.15) *vs* 2.40 (s.d. 2.09), *P* = 0.045] and pain [7.85 (s.d. 1.50) *vs* 5.10 (s.d. 2.52), *P* = 0.001], with a tendency for more tiredness [7.82 (s.d. 1.57) *vs* 6.31 (s.d. 2.70), *P* = 0.059] and waking up tired [7.94 (s.d. 1.79) *vs* 6.27 (s.d. 2.87), *P* = 0.059] (Table 3). With the ACR 2016 criteria questionnaire, all patients in the elevated hsCRP group fulfilled the criteria, although the proportion did not significantly differ from the normal hsCRP group (100% *vs* 82.8%, *P* = 0.555). WPI scores were similar in both groups, but the elevated hsCRP group had higher SSS scores [10.1 (s.d. 1.64) *vs* 7.11 (s.d. 2.28), *P* = 0.01]. For individual SSS items, the elevated hsCRP group reported more fatigue and cognitive impairment [moderate or severe 100% *vs* 62% (*P* = 0.024) and 87.5% *vs* 34.4% (*P* = 0.035), respectively], with a tendency for more depression (87.5% *vs* 44.8%, *P* = 0.101) and waking unrefreshed (moderate to severe, 75% *vs* 58.6%, *P* = 0.067) (Table 4). Finn-FIQ item 4 refers only to the ‘ability to work’, not the ‘ability to work, including housework’. As none in the elevated hsCRP group was working, only one patient reported the level of interference and thus no statistical test for this item could be conducted (Table 3). The elevated hsCRP group had slightly lower MCH (28.9 *vs* 30.4 pg/cell, *P* = 0.021) and MCHC (325 *vs* 335 g/l, *P* = 0.034) than the normal group, but did not differ significantly in blood Hb concentration (128 *vs* 134 g/l, *P* = 0.107) or in any other blood test result (Table 2 and Supplementary Table S2, available at *Rheumatology Advances in Practice* online).

**Table 3:** FIQ data for all FM patients, FM patients with normal hsCRP and FM patients with elevated hsCRP

Variables	All FM (*n* = 37)	Normal hsCRP (*n* = 29)	Elevated hsCRP (*n* = 8)
FIQ			
Mean (s.d.)	49.8 (18.9)	45.5 (17.4)	64.8 (17.2)
Median (minimum–maximum)	48.8 (12.2–89.5)	46.5 (12.2–78.4)	64.6 (37.2–89.5)
Missing, *n* (%)	1 (2.7)	1 (3.4)	0 (0)
*P*-value		**0.017**	
FIQ item 1: physical functioning			
Mean (s.d.)	2.81 (2.22)	2.40 (2.09)	4.33 (2.15)
Median (minimum–maximum)	2.33 (0.00–8.00)	1.67 (0–6.67)	4.67 (0.333–8.00)
*P*-value		**0.045**	
FIQ item 2: Of the 7 days in the past week, how many times did you feel good?			
Mean (s.d.)	2.67 (2.34)	2.67 (2.27)	2.67 (2.88)
Median (minimum–maximum)	3 (0–7)	3 (0–7)	2 (0–7)
Missing, *n* (%)	5 (13.5)	3 (10.3)	2 (25.0)
*P*-value		0.996	
FIQ item 3: days of missed work in the past week			
Mean (s.d.)	0.65 (1.74)	0.71 (1.81)	0 0
Median (minimum–maximum)	0 (0–7)	0 (0–7)	0 (0–0)
Missing, *n* (%)	11 (29.7)	5 (17.2)	6 (75.0)
*P*-value		0.067	
FIQ item 4: interference with ability to work			
Mean (s.d.)	4.75 (2.86)	4.77 (2.92)	4.15 (NA)
Median (minimum–maximum)	5.75 (0.00–9.04)	5.80 (0–9.043)	4.15 (4.15–4.15)
Missing, *n* (%)	14 (37.8)	7 (24.1)	7 (87.5)
*P*-value		**NA**	
FIQ item 5: pain			
Mean (s.d.)	5.71 (2.59)	5.10 (2.52)	7.85 (1.50)
Median (minimum–maximum)	6.38 (0.85–9.36)	5.16 (0.851–8.72)	8.56 (4.89–9.36)
Missing	1 (2.7)	1 (3.4)	0 (0)
*P*-value		**0.001**	
FIQ item 6: tiredness			
Mean (s.d.)	6.65 (2.55)	6.31 (2.70)	7.82 (1.57)
Median (minimum–maximum)	7.23 (1.38–9.89)	6.70 (1.38–9.89)	8.09 (4.89–9.79)
Missing, *n* (%)	1 (2.7)	1 (3.4)	0 (0)
*P*-value		0.059	
FIQ item 7: waking up tired			
Mean (s.d.)	6.64 (2.73)	6.27 (2.87)	7.94 (1.79)
Median (minimum–maximum)	7.39 (1.28–9.89)	7.13 (1.28–9.89)	8.67 (4.79–9.89)
Missing, *n* (%)	1 (2.7)	1 (3.4)	0 (0)
*P*-value		0.059	
FIQ item 8: stiffness			
Mean (s.d.)	6.37 (2.28)	6.15 (2.44)	7.14 (1.49)
Median (minimum–maximum)	6.81 (0.74–10.0)	6.44 (0.745–10.0)	6.86 (5.64–9.79)
Missing, *n* (%)	1 (2.7)	1 (3.4)	0 (0)
*P*-value		0.171	
FIQ item 9: nervousness or anxiety			
Mean (s.d.)	4.87 (2.89)	4.49 (2.81)	6.20 (2.98)
Median (minimum–maximum)	5.05 (0.11–10.4)	4.73 (0.106–9.89)	6.12 (1.38–10.4)
Missing, *n* (%)	1 (2.7)	1 (3.4)	0 (0)
*P*-value		0.175	
FIQ item 10: depression			
Mean (s.d.)	3.07 (2.61)	2.67 (2.53)	4.47 (2.53)
Median (minimum–maximum)	2.11 (0.00–9.79)	1.65 (0–8.25)	4.28 (1.55–9.79)
Missing, *n* (%)	1 (2.7)	1 (3.4)	0 (0)
*P*-value		0.102	

Statistical testing between the FM patient subgroups was done with a *t*-test for continuous variables and Fisher’s test for categorical variables. *P*-values <0.05 in bold.

**Table 4:** ACR 2016 criteria for FM data for all FM patients, FM patients with normal hsCRP and FM patients with elevated hsCRP

Criteria	All FM (*n* = 37)	Normal hsCRP (*n* = 29)	Elevated hsCRP (*n* = 8)
ACR 2016 diagnostic criteria fulfilled, *n* (%)			
Yes	32 (86.5)	24 (82.8)	8 (100)
No	4 (10.8)	4 (13.8)	0 (0)
Missing	1 (2.7)	1 (3.4)	0 (0)
*P*-value		0.555	
ACR 2016 SSS score			
Mean (s.d.)	7.78 (2.49)	7.11 (2.28)	10.1 (1.64)
Median (minimum–maximum)	7.50 (4.00–12.0)	7.00 (4.00–12.0)	10.5 (8.00–12.0)
Missing, *n* (%)	1 (2.7)	1 (3.4)	0 (0)
*P*-value		**<0.001**	
ACR 2016 WPI score			
Mean (s.d.)	11.1 (4.00)	10.5 (3.80)	13.0 (4.34)
Median (minimum–maximum)	11.0 (4.00–19.0)	11.0 (4.00–17.0)	12.5 (7.00–19.0)
Missing, *n* (%)	1 (2.7)	1 (3.4)	0 (0)
*P*-value		0.176	
ACR 2016 fatigue, *n* (%)			
1	10 (27.0)	10 (34.5)	0 (0)
2	13 (35.1)	11 (37.9)	2 (25.0)
3	13 (35.1)	7 (24.1)	6 (75.0)
Missing	1 (2.7)	1 (3.4)	0 (0)
*P*-value		**0.024**	
ACR 2016 waking unrefreshed, *n* (%)			
1	13 (35.1)	11 (37.9)	2 (25.0)
2	8 (21.6)	8 (27.6)	0 (0)
3	15 (40.5)	9 (31.0)	6 (75.0)
Missing	1 (2.7)	1 (3.4)	0 (0)
*P*-value		0.067	
ACR 2016 cognitive impairment, *n* (%)			
0	5 (13.5)	5 (17.2)	0 (0)
1	14 (37.8)	13 (44.8)	1 (12.5)
2	10 (27.0)	7 (24.1)	3 (37.5)
3	7 (18.9)	3 (10.3)	4 (50.0)
Missing	1 (2.7)	1 (3.4)	0 (0)
*P*-value		**0.035**	
ACR 2016 headache, *n* (%)			
No	4 (10.8)	3 (10.3)	1 (12.5)
Yes	32 (86.5)	25 (86.2)	7 (87.5)
Missing	1 (2.7)	1 (3.4)	0 (0)
*P*-value		1	
ACR 2016 depression, *n* (%)			
No	15 (40.5)	14 (48.3)	1 (12.5)
Yes	20 (54.1)	13 (44.8)	7 (87.5)
Missing	2 (5.4)	2 (6.9)	0 (0)
*P*-value		0.101	
ACR 2016 low abdominal pain, *n* (%)			
No	12 (32.4)	10 (34.5)	2 (25.0)
Yes	24 (64.9)	18 (62.1)	6 (75.0)
Missing	1 (2.7)	1 (3.4)	0 (0)
*P*-value		0.691	

Statistical testing between the FM patient subgroups was done with a *t*-test for continuous variables and Fisher’s test for categorical variables. *P*-values <0.05 in bold.

## Discussion

CRP is a widely used indicator of systemic inflammation. As with previous findings, we found FM patients to have slightly increased inflammation, shown by hsCRP, than controls. Adjusting for BMI and LTPA, FM patient status no longer explained differences in hsCRP, with higher BMI predicting higher hsCRP in both groups. HsCRP is increased by excessive adipose tissue and decreased in a dose-dependent manner by the frequency and intensity of LTPA [13, 14, 31]. While smoking has been associated with elevated hsCRP, our data did not confirm this [31].

Published research on CRP in FM is inconsistent, with six studies reporting elevated values compared with controls [12, 20, 22, 32–34] and three no difference [35–37]. Differences in CRP levels are largely explained by greater BMIs [20, 22, 34, 38]. Studies on ESR are also conflicting [22, 37, 39]. Levels of several cytokines are reported to be altered in FM—most consistently elevation of pro-inflammatory and reduction of anti-inflammatory cytokines [7, 38]. As these modulate immune responses in differing ways, the important factor in FM may be the combined pro/anti-inflammatory balance. CRP appears in two isoforms, pentameric, with anti-inflammatory properties, and monomeric, which is more pro-inflammatory [11]. As common assays of CRP do not differentiate between isoforms, there are few data on their relative roles in FM.

FM patients had higher LCs than controls, possibly indicating inflammation. Previous studies report both elevated LCs and no difference in FM patients compared with controls [35, 37]. In *post hoc* analysis, the group difference in LCs was explained by adjusting for BMI, LTPA and smoking. BMI and smoking predicted higher LCs and LTPA predicted lower LCs, with smoking having the greatest effect, consistent with the known effects of these lifestyle factors on LCs [40, 41]. LCs correlated with FIQ scores but not with hsCRP levels. As we did not differentiate leucocyte types in our study, the significance of higher LCs in FM patients in our study remains unknown.

FM patients with elevated hsCRP had lower MCH and MCHC, with a tendency for lower blood Hb concentrations. These findings resemble changes seen in anaemia associated with chronic diseases, in which long-term systemic inflammation leads to reduced erythropoiesis and hypochromic erythrocytes [42]. These changes are not specific for FM but may play a role in the worsening of symptoms.

Why do some FM patients present elevated levels of CRP in greater frequency than the healthy population? Some of this variation is likely to be genetic. Xiao *et al.* [21] found that FM patients carrying the Val66Val polymorphism of the brain-derived neurotrophic factor (BDNF) were more likely to have both higher BMI and elevated hsCRP than FM patients with Val66Met. Controls did not have this connection between hsCRP, BMI and BDNF polymorphism. Low levels of BDNF are linked to cognitive decline and depression, with circulating levels increased by physical exercise [43]. We may hypothesize that FM symptoms of cognitive impairment and depressive mood are mediated by a lack of BDNF.

Interestingly, Goebel *et al.* [44] reported recently that they were able to cause FM-like hyperalgesia with reduced locomotor activity and paw grip strength in mice with a transfusion of plasma from FM patients having severe symptoms but not from controls. These symptoms may associate with IgG antibodies targeting dorsal root ganglia neurons and satellite glial cells. While this possible autoimmune link to FM is yet to be verified, it would seem logical that inflammatory activity could increase humoral activity and that inflammation caused by overweight and low physical activity could be linked to worsened FM symptoms.

In our study, FM patients with elevated hsCRP had higher FIQ and ACR 2016 symptom severity scores, consistent with previous findings [23, 33]. Rus *et al.* [33] found this correlation only in overweight patients, likely due to the fact that they had higher CRP. When analysing individual symptoms, the elevated hsCRP group reported worse physical functioning, pain, fatigue and cognitive symptoms and showed a tendency to report more tiredness, depression and waking unrefreshed. We saw no difference in stiffness, anxiety or WPI score. Surprisingly, the FM patients in the elevated hsCRP group were far more likely to be outside working life. We did not collect data on why this was, but this suggests a greater impact of their (inflammation-associated) worsened symptoms. We may hypothesize that not just pain and impaired physical functioning, but also cognitive symptoms connected to low-grade inflammation, high BMI and unrefreshing sleep combined with the high cognitive demands of modern working life could contribute to this finding.

Insomnia is linked to elevated CRP [45] and high BMI has been associated with more tender points and greater fatigue and tiredness in FM patients [46]. As high BMI is a significant risk factor for sleep apnoea, the latter is one possible mechanism connecting high BMI, unrefreshing sleep and the symptoms described above. Sleep apnoea [47] may be more prevalent in FM patients, although this needs verification. As the burden of FM symptoms is a risk factor for premature retirement, unravelling this would be of great socio-economic importance [48].

## Strengths and limitations

Our study has an adequately sized sample of FM patients whose disease severity closely resembles that encountered by most physicians. We did not include male patients. Thus gender effects were not examined and the generalizability of our results to male patients is limited. There was a delay between collecting questionnaire data and blood samples, due to the demands of a protocol that had to fit several laboratory tests into participants’ timetables. The median time gap was 86 days (interquartile range 30–298). Thus there may have been time for changes in questionnaire scores as blood samples were collected, which would affect correlation analyses and subgroup comparisons. However, FM symptoms seem to be stable over long periods of time, even decades, suggesting stability in symptom severity subgroups [49]. Of the other questionnaires, the STAI-B measures anxiety traits that are, by definition, stable, while the PCS measures both state and trait catastrophizing, with scores seeming relatively stable, at least over a few months [50, 51]. Sleep quality has been linked to inflammation, but we could not confirm this [45], perhaps due to the lack of a validated sleep questionnaire.

## Conclusion

Low-grade inflammation detected by hsCRP is present in FM and correlates with symptom severity. Elevated hsCRP is more likely explained by overweight and low physical activity than by FM. Some FM patients may be particularly vulnerable to low-grade inflammation and associated worsening of symptoms, possibly leading to impaired working ability. Thus the role of pharmacotherapy targeting the inflammatory system in FM warrants further research. Physical activity and normal weight are recommended for all FM patients, but hsCRP may help identify those at the greatest risk who would benefit most from lifestyle interventions. The importance of lifestyle factors is supported by the positive correlation between BMI and hsCRP and changes indicative of metabolic syndrome seen in FM patients with elevated hsCRP.

## Supplementary Material

rkac053_Supplementary_Data
