# Supplementary material for: Elevated highly sensitive C-reactive protein in fibromyalgia associates with symptom severity
Source: Rheumatol Adv Pract. 2022 Jun 25;6(2):rkac053. doi: 10.1093/rap/rkac053 (PMC9272915; doi:10.1093/rap/rkac053)
Supplement: rkac053_Supplementary_Data [file rkac053_supplementary_data.zip › ZettermanSupplTable2.docx]

**Supplementary Table S2: Full blood sample data for healthy controls, all FM patients, FM patients with normal hsCRP, and FM patients with elevated hsCRP.**

|  | **Control (n = 29)** | **FM (n = 37)** | **FM (normal hsCRP) (n = 29)** | **FM (elevated hsCRP) (n = 8)** |
| --- | --- | --- | --- | --- |
| **Highly sensitive C-reactive protein (mg/l)** |  |  |  |  |
| Mean (SD) | 1.17 (1.11) | 2.33 (2.43) | 1.19 (0.675) | 6.46 (1.89) |
| Median [Min, Max] | 0.680 [0.130, 3.78] | 1.51 [0.190, 9.22] | 1.12 [0.190, 2.66] | 6.46 [3.30, 9.22] |
| p-value | **0.0128** | | **< 0.001** | |
| **Elevated hsCRP** |  |  |  |  |
| No | 26 (89.7%) | 29 (78.4%) | 29 (100%) | 0 (0%) |
| Yes | 3 (10.3%) | 8 (21.6%) | 0 (0%) | 8 (100%) |
| p-value | 0.323 | | **< 0.001** | |
| **Haemoglobin (g/l)** |  |  |  |  |
| Mean (SD) | 128 (7.59) | 133 (7.77) | 134 (7.09) | 128 (8.74) |
| Median [Min, Max] | 128 [105, 145] | 132 [115, 153] | 133 [121, 153] | 130 [115, 140] |
| Missing | 0 (0%) | 1 (2.7%) | 1 (3.4%) | 0 (0%) |
| p-value | **0.021** | | 0.107 | |
| **Haematocrit (%)** |  |  |  |  |
| Mean (SD) | 38.8 (2.08) | 40.0 (2.32) | 40.1 (2.22) | 39.5 (2.73) |
| Median [Min, Max] | 39.0 [34.0, 44.0] | 40.0 [36.0, 44.0] | 40.0 [36.0, 44.0] | 39.5 [36.0, 43.0] |
| Missing | 0 (0%) | 1 (2.7%) | 1 (3.4%) | 0 (0%) |
| p-value | **0.0266** | | 0.555 | |
| **Erythrocyte count (10^12^/l)** |  |  |  |  |
| Mean (SD) | 4.36 (0.276) | 4.45 (0.329) | 4.43 (0.308) | 4.48 (0.419) |
| Median [Min, Max] | 4.30 [3.76, 5.02] | 4.40 [3.95, 5.31] | 4.40 [3.95, 5.12] | 4.39 [4.03, 5.31] |
| Missing | 0 (0%) | 1 (2.7%) | 1 (3.4%) | 0 (0%) |
| p-value | 0.284 | | 0.77 | |
| **Mean cellular volume of erythrocytes (fl)** |  |  |  |  |
| Mean (SD) | 88.7 (4.24) | 90.0 (4.18) | 90.5 (4.24) | 88.3 (3.69) |
| Median [Min, Max] | 89.0 [75.0, 95.0] | 90.0 [80.0, 97.0] | 91.0 [81.0, 97.0] | 89.0 [80.0, 93.0] |
| Missing | 0 (0%) | 1 (2.7%) | 1 (3.4%) | 0 (0%) |
| p-value | 0.208 | | 0.16 | |
| **Red cell distribution width (%)** |  |  |  |  |
| Mean (SD) | 12.8 (1.26) | 13.1 (0.754) | 13.0 (0.793) | 13.1 (0.641) |
| Median [Min, Max] | 12.0 [11.0, 17.0] | 13.0 [12.0, 14.0] | 13.0 [12.0, 14.0] | 13.0 [12.0, 14.0] |
| Missing | 0 (0%) | 1 (2.7%) | 1 (3.4%) | 0 (0%) |
| p-value | 0.33 | | 0.747 | |
| **Mean corpuscular haemoglobin (pg)** |  |  |  |  |
| Mean (SD) | 29.5 (2.03) | 30.0 (1.63) | 30.4 (1.57) | 28.9 (1.36) |
| Median [Min, Max] | 30.0 [23.0, 33.0] | 30.0 [26.0, 34.0] | 31.0 [26.0, 34.0] | 29.0 [26.0, 30.0] |
| Missing | 0 (0%) | 1 (2.7%) | 1 (3.4%) | 0 (0%) |
| p-value | 0.246 | | **0.0209** | |
| **Mean corpuscular haemoglobin concentration (g/l)** |  |  |  |  |
| Mean (SD) | 332 (8.63) | 332 (9.22) | 335 (7.74) | 325 (10.4) |
| Median [Min, Max] | 332 [309, 350] | 333 [311, 349] | 334 [311, 349] | 321 [311, 344] |
| Missing | 0 (0%) | 1 (2.7%) | 1 (3.4%) | 0 (0%) |
| p-value | 0.707 | | **0.0342** | |
| **Leukocyte count (10^9^/l)** |  |  |  |  |
| Mean (SD) | 4.99 (1.10) | 6.06 (1.77) | 5.97 (1.64) | 6.35 (2.27) |
| Median [Min, Max] | 5.00 [3.00, 8.80] | 6.10 [3.00, 10.8] | 5.90 [3.20, 10.0] | 6.45 [3.00, 10.8] |
| Missing | 0 (0%) | 1 (2.7%) | 1 (3.4%) | 0 (0%) |
| p-value | **0.00439** | | 0.67 | |
| **Thrombocyte count (10^9^/l)** |  |  |  |  |
| Mean (SD) | 261 (41.8) | 276 (65.5) | 275 (62.9) | 279 (78.7) |
| Median [Min, Max] | 259 [196, 342] | 271 [170, 469] | 268 [176, 469] | 286 [170, 392] |
| Missing | 0 (0%) | 1 (2.7%) | 1 (3.4%) | 0 (0%) |
| p-value | 0.264 | | 0.907 | |
| **Activated partial thromboplastin time (s)** |  |  |  |  |
| Mean (SD) | 29.1 (2.31) | 28.9 (2.17) | 28.8 (2.23) | 29.3 (2.05) |
| Median [Min, Max] | 29.0 [25.0, 33.0] | 29.0 [25.0, 34.0] | 28.0 [25.0, 34.0] | 29.0 [27.0, 33.0] |
| p-value | 0.661 | | 0.594 | |
| **International normalized ratio** |  |  |  |  |
| Mean (SD) | 0.997 (0.115) | 0.957 (0.0899) | 0.969 (0.0930) | 0.913 (0.0641) |
| Median [Min, Max] | 1.00 [0.900, 1.40] | 0.900 [0.800, 1.20] | 1.00 [0.800, 1.20] | 0.900 [0.800, 1.00] |
| p-value | 0.131 | | 0.0648 | |
| **Creatine kinase (U/l)** |  |  |  |  |
| Mean (SD) | 119 (111) | 99.2 (56.3) | 97.3 (55.8) | 106 (61.6) |
| Median [Min, Max] | 84.0 [45.0, 650] | 81.0 [38.0, 219] | 75.0 [38.0, 209] | 90.5 [40.0, 219] |
| p-value | 0.383 | | 0.726 | |
| **Total cholesterol (mmol/l)** |  |  |  |  |
| Mean (SD) | 4.84 (0.766) | 5.38 (0.945) | 5.28 (0.967) | 5.74 (0.812) |
| Median [Min, Max] | 5.00 [3.10, 6.40] | 5.30 [3.60, 7.40] | 5.20 [3.60, 7.40] | 5.60 [4.40, 6.80] |
| p-value | **0.0142** | | 0.196 | |
| **High density lipoprotein (HDL) (mmol/l)** |  |  |  |  |
| Mean (SD) | 1.72 (0.370) | 1.59 (0.376) | 1.59 (0.343) | 1.59 (0.509) |
| Median [Min, Max] | 1.72 [1.05, 2.65] | 1.61 [0.900, 2.47] | 1.61 [0.900, 2.29] | 1.62 [0.920, 2.47] |
| p-value | 0.186 | | 0.98 | |
| **Low density lipoprotein (mmol/l)** |  |  |  |  |
| Mean (SD) | 2.74 (0.571) | 3.42 (0.869) | 3.38 (0.861) | 3.59 (0.937) |
| Median [Min, Max] | 2.70 [1.50, 3.80] | 3.40 [1.80, 5.10] | 3.30 [2.10, 5.10] | 3.65 [1.80, 4.90] |
| p-value | **< 0.001** | | 0.583 | |
| **Triglycerides (mmol/l)** |  |  |  |  |
| Mean (SD) | 0.974 (0.661) | 1.28 (0.665) | 1.27 (0.732) | 1.34 (0.355) |
| Median [Min, Max] | 0.770 [0.440, 3.73] | 1.08 [0.530, 3.64] | 1.03 [0.530, 3.64] | 1.38 [0.820, 1.76] |
| p-value | 0.0635 | | 0.687 | |
| **Glucose at 0h (mmol/l)** |  |  |  |  |
| Mean (SD) | 5.31 (0.501) | 5.45 (0.535) | 5.42 (0.538) | 5.56 (0.542) |
| Median [Min, Max] | 5.30 [4.30, 6.40] | 5.40 [4.50, 6.80] | 5.30 [4.50, 6.80] | 5.55 [4.70, 6.30] |
| p-value | 0.263 | | 0.525 | |
| **Glucose AUC (mmol*h/l)** |  |  |  |  |
| Mean (SD) | 11.4 (2.16) | 13.7 (2.95) | 13.8 (3.21) | 13.1 (1.81) |
| Median [Min, Max] | 11.2 [7.55, 15.2] | 13.1 [8.70, 20.6] | 13.7 [8.70, 20.6] | 12.6 [11.2, 16.7] |
| Missing | 3 (10.3%) | 2 (5.4%) | 2 (6.9%) | 0 (0%) |
| p-value | **0.00118** | | 0.39 | |
| **Impaired glucose regulation** |  |  |  |  |
| Yes | 5 (17.2%) | 14 (37.8%) | 11 (37.9%) | 3 (37.5%) |
| No | 24 (82.8%) | 23 (62.2%) | 18 (62.1%) | 5 (62.5%) |
| p-value | 0.1 | | 1 | |
| **Lactate at 0h (mmol/l)** |  |  |  |  |
| Mean (SD) | 1.44 (2.30) | 0.970 (0.270) | 0.943 (0.268) | 1.08 (0.271) |
| Median [Min, Max] | 0.900 [0.600, 12.4] | 1.00 [0.600, 1.70] | 0.900 [0.600, 1.70] | 1.10 [0.800, 1.50] |
| Missing | 4 (13.8%) | 6 (16.2%) | 4 (13.8%) | 2 (25.0%) |
| p-value | 0.32 | | 0.29 | |
| **Lactate AUC (mmol*h/l)** |  |  |  |  |
| Mean (SD) | 1.43 (1.16) | 1.18 (0.238) | 1.17 (0.240) | 1.23 (0.250) |
| Median [Min, Max] | 1.20 [0.750, 6.70] | 1.15 [0.750, 1.75] | 1.15 [0.750, 1.75] | 1.25 [0.900, 1.50] |
| Missing | 6 (20.7%) | 9 (24.3%) | 5 (17.2%) | 4 (50.0%) |
| p-value | 0.32 | | 0.716 | |
| **Pyruvate at 0h (μmol/l)** |  |  |  |  |
| Mean (SD) | 92.8 (16.0) | 92.2 (13.7) | 92.9 (12.8) | 89.4 (17.3) |
| Median [Min, Max] | 93.0 [61.0, 133] | 91.0 [66.0, 122] | 92.0 [69.0, 122] | 86.5 [66.0, 121] |
| Missing | 1 (3.4%) | 0 (0%) |  |  |
| p-value | 0.877 | | 0.601 | |
| **Pyruvate AUC (μmol*h/l)** |  |  |  |  |
| Mean (SD) | 97.9 (14.2) | 96.3 (11.3) | 97.4 (11.5) | 91.7 (10.1) |
| Median [Min, Max] | 98.5 [67.0, 134] | 95.0 [73.5, 124] | 95.5 [77.0, 124] | 94.8 [73.5, 103] |
| Missing | 4 (13.8%) | 4 (10.8%) | 2 (6.9%) | 2 (25.0%) |
| p-value | 0.661 | | 0.257 | |
| **pH** |  |  |  |  |
| Mean (SD) | 7.40 (0.0246) | 7.40 (0.0280) | 7.40 (0.0292) | 7.41 (0.0256) |
| Median [Min, Max] | 7.40 [7.35, 7.45] | 7.40 [7.35, 7.45] | 7.39 [7.35, 7.45] | 7.40 [7.37, 7.45] |
| Missing | 8 (27.6%) | 8 (21.6%) | 8 (27.6%) | 0 (0%) |
| p-value | 0.597 | | 0.61 | |
| **Actual ionized calcium (mmol/l)** |  |  |  |  |
| Mean (SD) | 1.20 (0.0464) | 1.21 (0.0293) | 1.21 (0.0303) | 1.21 (0.0285) |
| Median [Min, Max] | 1.19 [1.12, 1.30] | 1.21 [1.15, 1.27] | 1.21 [1.15, 1.27] | 1.22 [1.17, 1.24] |
| Missing | 8 (27.6%) | 8 (21.6%) | 8 (27.6%) | 0 (0%) |
| p-value | 0.813 | | 0.805 | |
| **Normalized ionized calcium (mmol/l/pH7.4)** |  |  |  |  |
| Mean (SD) | 1.20 (0.0417) | 1.21 (0.0266) | 1.21 (0.0289) | 1.21 (0.0203) |
| Median [Min, Max] | 1.19 [1.10, 1.28] | 1.21 [1.15, 1.25] | 1.21 [1.15, 1.25] | 1.22 [1.18, 1.24] |
| Missing | 8 (27.6%) | 8 (21.6%) | 8 (27.6%) | 0 (0%) |
| p-value | 0.601 | | 0.537 | |
| **Alanine transaminase (U/l)** |  |  |  |  |
| Mean (SD) | 21.0 (13.6) | 24.0 (14.4) | 25.8 (16.4) | 19.3 (5.15) |
| Median [Min, Max] | 17.0 [8.00, 66.0] | 22.0 [9.00, 71.0] | 22.0 [9.00, 71.0] | 17.5 [13.0, 27.0] |
| Missing | 7 (24.1%) | 8 (21.6%) | 8 (27.6%) | 0 (0%) |
| p-value | 0.457 | | 0.115 | |
| **Alkaline phosphatase (U/l)** |  |  |  |  |
| Mean (SD) | 64.2 (18.3) | 67.0 (17.8) | 66.3 (18.8) | 68.9 (15.9) |
| Median [Min, Max] | 59.0 [42.0, 112] | 66.0 [41.0, 116] | 65.0 [41.0, 116] | 70.0 [43.0, 90.0] |
| Missing | 7 (24.1%) | 8 (21.6%) | 8 (27.6%) | 0 (0%) |
| p-value | 0.584 | | 0.716 | |
| **Aspartate transaminase (U/l)** |  |  |  |  |
| Mean (SD) | 23.6 (4.31) | 25.4 (10.9) | 24.5 (7.58) | 28.0 (17.2) |
| Median [Min, Max] | 23.0 [17.0, 36.0] | 22.0 [14.0, 69.0] | 24.0 [14.0, 40.0] | 21.5 [17.0, 69.0] |
| Missing | 7 (24.1%) | 8 (21.6%) | 8 (27.6%) | 0 (0%) |
| p-value | 0.407 | | 0.592 | |
| **Anti-nuclear antibodies** |  |  |  |  |
| neg | 20 (69.0%) | 27 (73.0%) | 19 (65.5%) | 8 (100%) |
| pos | 1 (3.4%) | 2 (5.4%) | 2 (6.9%) | 0 (0%) |
| Missing | 8 (27.6%) | 8 (21.6%) | 8 (27.6%) | 0 (0%) |
| p-value | 1 | | 1 | |
| **Extractable nuclear antigens** |  |  |  |  |
| neg | 20 (69.0%) | 28 (75.7%) | 20 (69.0%) | 8 (100%) |
| pos | 1 (3.4%) | 1 (2.7%) | 1 (3.4%) | 0 (0%) |
| Missing | 8 (27.6%) | 8 (21.6%) | 8 (27.6%) | 0 (0%) |
| p-value | 1 | | 1 | |
| **Glutamate decarboxylase antibodies** |  |  |  |  |
| neg | 21 (72.4%) | 29 (78.4%) | 21 (72.4%) | 8 (100%) |
| pos | 0 (0%) | 0 (0%) | 0 (0%) | 0 (0%) |
| Missing | 8 (27.6%) | 8 (21.6%) | 8 (27.6%) | 0 (0%) |
| p-value | 1 | | 1 | |

Statistical testing between healthy controls and all FM patients, and between FM patient subgroups was done with t-test for continuous variables and Fisher’s test for categorical variables. hsCRP: highly sensitive C-reactive protein. P-values < 0.05 in bold.
