# Supplementary material for: Elevated highly sensitive C-reactive protein in fibromyalgia associates with symptom severity
Source: Rheumatol Adv Pract. 2022 Jun 25;6(2):rkac053. doi: 10.1093/rap/rkac053 (PMC9272915; doi:10.1093/rap/rkac053)
Supplement: rkac053_Supplementary_Data [file rkac053_supplementary_data.zip › ZettermanSupplTable1.docx]

**Supplementary Table S1: Comorbidities of healthy controls, all FM patients, FM patients with normal hsCRP, and FM patients with elevated hsCRP.**

|  | **Control  (n = 29)** | **FM (n = 37)** | **FM (normal hsCRP) (n = 29)** | **FM (elevated hsCRP) (n = 8)** |
| --- | --- | --- | --- | --- |
| **Number of comorbidities** |  |  |  |  |
| Mean (SD) | 0.50 (0.66) | 2.08 (1.81) | 1.82 (1.68) | 3.00 (2.07) |
| Median [Min, Max] | 0 [0, 2.00] | 2.00 [0, 7.00] | 2.00 [0, 5.00] | 2.50 [1.00, 7.00] |
| Missing | 5 (17.2%) | 1 (2.7%) | 1 (3.4%) | 0 (0%) |
| p-value | **< 0.001** | | 0.171 | |
| **Cardiovascular disease^1^** |  |  |  |  |
| No | 29 (100%) | 35 (94.6%) | 27 (93.1%) | 8 (100%) |
| Yes | 0 (0%) | 2 (5.4%) | 2 (6.9%) | 0 (0%) |
| p-value | 0.5 | | 1 | |
| **Endocrinological disease^2^** |  |  |  |  |
| No | 28 (96.6%) | 31 (83.8%) | 25 (86.2%) | 6 (75.0%) |
| Yes | 1 (3.4%) | 6 (16.2%) | 4 (13.8%) | 2 (25.0%) |
| p-value | 0.124 | | 0.591 | |
| **Musculoskeletal disease^3^** |  |  |  |  |
| No | 27 (93.1%) | 27 (73.0%) | 23 (79.3%) | 4 (50.0%) |
| Yes | 2 (6.9%) | 10 (27.0%) | 6 (20.7%) | 4 (50.0%) |
| p-value | 0.053 | | 0.174 | |
| **Neurological disease^4^** |  |  |  |  |
| No | 24 (82.8%) | 24 (64.9%) | 19 (65.5%) | 5 (62.5%) |
| Yes | 5 (17.2%) | 13 (35.1%) | 10 (34.5%) | 3 (37.5%) |
| p-value | 0.206 | | 1 | |
| **Psychiatric disease^5^** |  |  |  |  |
| No | 29 (100%) | 32 (86.5%) | 25 (86.2%) | 7 (87.5%) |
| Yes | 0 (0%) | 5 (13.5%) | 4 (13.8%) | 1 (12.5%) |
| p-value | 0.062 | | 1 | |
| **Pulmonary disease^6^** |  |  |  |  |
| No | 27 (93.1%) | 30 (81.1%) | 25 (86.2%) | 5 (62.5%) |
| Yes | 2 (6.9%) | 7 (18.9%) | 4 (13.8%) | 3 (37.5%) |
| p-value | 0.279 | | 0.156 | |
| **Rheumatic disease^7^** |  |  |  |  |
| No | 29 (100%) | 34 (91.9%) | 7 (87.5%) | 27 (93.1%) |
| Yes | 0 (0%) | 3 (8.1%) | 1 (12.5%) | 2 (6.9%) |
| p-value | 0.25 | | 0.53 | |
| **Other comorbidity^8^** |  |  |  |  |
| No | 27 (93.1%) | 24 (64.9%) | 20 (69.0%) | 4 (50.0%) |
| Yes | 2 (6.9%) | 13 (35.1%) | 9 (31.0%) | 4 (50.0%) |
| p-value | **0.008** | | 0.413 | |

Statistical testing between healthy controls and all FM patients, and between FM patient subgroups was done with Fisher’s test. Individual diagnoses in each category. Number of cases (n) = 1 if not otherwise noted. ^1^hypertension or paroxysmal tachycardia; ^2^hypercholesterolaemia or hypothyroidism (n = 5); ^3^cervical pain, Ehlers-Danlos syndrome, hip dysplasia, other hip condition, lumbar radiculopathy, osteoarthrosis (n = 5), sequela of elbow injury, spinal stenosis, or vertebral condition (n = 4); ^4^essential tremor, meralgia paraesthetica, migraine (n = 15), or trigeminal neuralgia (n = 2); ^5^ bipolar disorder or depression (n = 4); ^6^asthma (n = 8) or sleep apnoea; ^7^rheumatoid arthritis (n = 3), spondyloarthritis (n = 2); ^8^ allergy (n = 3), atopic eczema, Crohn’s disease, chronic fatigue syndrome, chronic rhinitis, coeliac disease, collagen colitis, Dubin-Johnson syndrome, gastro-oesophageal reflux (n = 6), interstitial cystitis, irritable bowel syndrome (n = 2), microscopic lymphatic colitis, multiple chemical sensitivity, myopia, polycystic ovary syndrome, Raynaud’s syndrome, Sjögren syndrome, urticaria, vulvodynia.

hsCRP: highly sensitive C-reactive protein. P-values < 0.05 in bold.
